# The Effect of Liposomal DMU-212 on the Differentiation of Human Ovarian Granulosa Cells in a Primary 3D Culture Model

**DOI:** 10.3390/ph18101460

**Published:** 2025-09-28

**Authors:** Małgorzata Jόzkowiak, Dariusz Wawrzyniak, Alicja Kawczyńska, Paulina Skupin-Mrugalska, Mikołaj Czajkowski, Paul Mozdziak, Marta Podralska, Marek Żywicki, Bartosz Kempisty, Robert Z. Spaczyński, Hanna Piotrowska-Kempisty

**Affiliations:** 1 Department of Toxicology, Poznan University of Medical Sciences, 60-806 Poznan, Poland; mjozkowiak@ump.edu.pl (M.J.); alicjakawczynska26@gmail.com (A.K.); 2 Doctoral School, Poznan University of Medical Sciences, 60-812 Poznan, Poland; 3 Department of Molecular Neurooncology, Institute of Bioorganic Chemistry, Polish Academy of Sciences, 61-704 Poznan, Poland; 4 Department of Inorganic & Analytical Chemistry, Poznan University of Medical Sciences, 60-806 Poznan, Poland; 5 Prestage Department of Poultry Science, North Carolina State University, Raleigh, NC 27695, USA; pemozdzi@ncsu.edu; 6 Physiology Graduate Faculty, North Carolina State University, Raleigh, NC 27613, USA; 7 Department of Stem Cells and Regenerative Medicine, Institute of Natural Fibres and Medicinal Plants, 62-064 Plewiska, Poland; 8 Department of Computational Biology, Institute of Molecular Biology, Faculty of Biology, Adam Mickiewicz University, 61-614 Poznan, Poland; 9 Division of Anatomy, Department of Human Morphology and Embryology, Wroclaw Medical University, 50-368 Wroclaw, Poland; 10 Department of Veterinary Surgery, Institute of Veterinary Medicine, Nicolaus Copernicus University in Torun, 87-100 Torun, Poland; 11 Department of Obstetrics and Gynecology, University Hospital and Masaryk University, 62500 Brno, Czech Republic; 12 Center for Gynecology, Obstetrics and Infertility Treatment Pastelova, 60-198 Poznan, Poland; 13 Collegium Medicum, University of Zielona Gora, 65-046 Zielona Gora, Poland; 14 Department of Basic and Preclinical Sciences, Institute of Veterinary Medicine, Nicolaus Copernicus University in Torun, 87-100 Torun, Poland

**Keywords:** 3,4,5,4′-tetramethoxystilbene (DMU-212), granulosa cells, differentiation, osteoblasts, RNA-seq

## Abstract

**Background/Objectives:** Human ovarian granulosa cells (hGCs) are crucial to ovarian follicle development and function, exhibiting multipotency and the ability to differentiate into neuronal cells, chondrocytes, and osteoblasts in vitro. 3,4,5,4′-tetramethoxystilbene (DMU-212) is a methylated derivative of resveratrol, a natural polyphenol found in grapes and berries, with a wide spectrum of biological activities, including notable anticancer properties. Interestingly, DMU-212 exhibits cytotoxic effects predominantly on cancer cells while sparing non-cancerous ones, and evidence suggests that similar to resveratrol, it may also promote hGC differentiation. This study aimed to investigate the effects of the liposomal formulation of this methylated resveratrol analog—lipDMU-212—on the osteogenic differentiation ability of hGCs in a primary three-dimensional cell culture model. **Methods:** lipDMU-212 was formulated using the thin-film hydration method. GC spheroids’ viability was evaluated after exposure to lipDMU-212, an osteoinductive medium, or both. Osteogenic differentiation was confirmed using Alizarin Red staining and quantified by measuring Alkaline Phosphatase (ALP) activity on days 1, 7, and 15. RNA sequencing (RNA-seq) was performed to explore molecular mechanisms underlying lipDMU-212-induced differentiation. **Results:** lipDMU-212 promoted osteogenic differentiation of hGCs in the 3D cell culture model, as evidenced by increased mineralization and a ~4-fold increase in ALP activity compared with the control. RNA-seq revealed up-regulation of genes related to cell differentiation and cellular identity. Furthermore, *JUN* (+2.82, *p* = 0.003), *LRP1* (+2.06, *p* = 0.05), *AXIN1* (+3.02, *p* = 0.03), and *FYN* (+3.30, *p* = 0.01) were up-regulated, indicating modulation of the Wnt/β-catenin signaling pathway, a key regulator of osteoblast differentiation. **Conclusions:** The ability of GCs to differentiate into diverse tissue-specific cell types underscores their potential in regenerative medicine. This study contributes to the understanding of lipDMU-212’s role in osteogenic differentiation and highlights its potential in developing future therapies for degenerative bone diseases.

## 1. Introduction

Age-related bone loss increases fracture risk in elderly individuals. This process is attributed to a shift in bone tissue composition, with decreased osteogenesis and elevated bone marrow fat accumulation [1,2,3]. The ability to effectively regenerate bone tissue is crucial to the treatment of osteoporosis, fractures, and other bone diseases. Therefore, scientific research increasingly focuses on stem cells as a promising approach to promoting osteoblast differentiation and enhancing bone regeneration.

hGCs, along with theca cells, form a structural component of the ovarian follicle and play a crucial role in steroidogenesis, particularly in the production of estradiol and progesterone. Recent studies have indicated that hGCs exhibit stem cell-like properties, as evidenced by the expression of Oct-4, CD29, CD44, CD90, CD105, CD117, CD166, Nanog, and Sox-2 markers [4,5]. To date, it has been observed that hGCs can differentiate into neuronal cells, osteoblasts, and chondroblasts when exposed to specific factors or compounds, such as leukemia-inhibiting factor (LIF) or follicle-stimulating hormone (FSH) [6,7,8].

Resveratrol (3,4′,5-trans-trihydroxystilbene) is a naturally occurring polyphenol that is widely known for its extraordinary properties, including antioxidant, anti-inflammatory, and anticancer ones. Moreover, resveratrol and its derivatives are also recognized for their ability to promote the differentiation of stem cells into various specialized cell lines [reviewed in [9]]. For instance, polydatin, a precursor of resveratrol, was found to enhance the osteogenic differentiation of dental bud stem cells [10]. As demonstrated in numerous studies, resveratrol has also been shown to support ovarian function, stimulate the development of ovarian follicles, prevent GCs from cell degeneration, and promote their proliferation and differentiation [11,12,13,14]. Despite the relatively high absorption of resveratrol, its rapid metabolism and elimination from the circulation lead to a bioavailability of less than 1% [15].

The substitution of hydroxyl (-OH) groups with methoxy (-OMe) ones at the −3, −5 positions of resveratrol has been shown to enhance the biological activity of its analogs. One of such resveratrol’s methoxy derivatives is DMU-212. This compound evokes enhanced anticancer activity, including apoptosis induction and inhibition of cell growth, compared with resveratrol. Sale et al. showed that unlike resveratrol, DMU-212 undergoes metabolism through hydroxylation and single or double O-demethylation. After administration, DMU-212 levels in the brain and in the small and large intestinal mucosa were higher than those of resveratrol, indicating improved blood–brain barrier penetration due to its increased lipophilicity [16]. Sale et al.’s findings are in line with structure–activity relationship studies since they indicate that methoxy groups added to the resveratrol backbone enhance its lipophilicity, thereby increasing cellular uptake. Concomitantly, introducing methoxy groups to the stilbene backbone has also been shown to decrease the compound’s solubility in an aqueous environment [17]. As a result, DMU-212 exhibits poor solubility in cell culture media. To address this limitation, we employed liposomal delivery systems, which are capable of encapsulating molecules with unfavorable solubility profiles while maintaining compatibility with both lipophilic and hydrophilic compounds. Furthermore, studies by Nowicki et al. have shown that another methylated derivative of resveratrol, DMU-214, exhibits enhanced biological activity when administered in a liposomal formulation compared with the free compound [18].

DMU-212 has been revealed to enhance estradiol and progesterone production in hGCs due to an increase in transcript levels of steroidogenesis-related genes, especially *StAR* and *CYP19A1*, which encode proteins crucial to estradiol secretion [19]. In view of these data and the structural similarity of DMU-212 to resveratrol, it could also be suggested to influence hGC differentiation into different cell lineages. Given the progressively aging population and the increasing prevalence of bone diseases, it is crucial to explore new, advanced strategies and bioactive compounds that could support the therapeutic process.

In light of these findings, this study aimed to evaluate the impact of the liposomal formulation of DMU-212 on the osteogenic differentiation ability of hGCs in a primary three-dimensional cell culture model.

## 2. Results

### 2.1. Characterization of lipDMU-212

**Figure 1:**
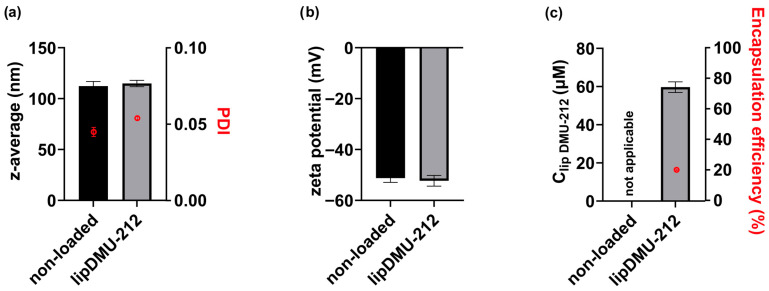
Characteristics of non-loaded and DMU-212-loaded liposomes (lipDMU-212): (**a**) size expressed as z-average (nm) and size distribution (polydispersity index, PDI), (**b**) zeta potential (mV), and (**c**) DMU-212 concentration in liposomes and encapsulation efficacy (%).

The characteristics of lipDMU-212 and non-loaded liposomes are shown in Figure 1. The average particle size (z-average) of lipDMU-212 was 114.9 ± 3.1 nm, which did not differ significantly from that of unloaded liposomes (112.3 ± 4.6 nm). The PDI value for lipDMU-212 was 0.054 ± 0.001, whereas for unloaded liposomes, it was 0.045 ± 0.003. Due to the presence of POPG, the liposomal membranes carried a negative surface charge, with zeta potentials of −51.2 mV for unloaded liposomes and −52.3 mV for lipDMU-212. HPLC analysis revealed a DMU-212 concentration of 59.7 ± 2.8 µM in lipDMU-212, corresponding to approximately 20% encapsulation efficiency. For subsequent experiments, lipDMU-212 liposomes were diluted in cell culture medium.

### 2.2. Cell Viability

**Figure 2:**
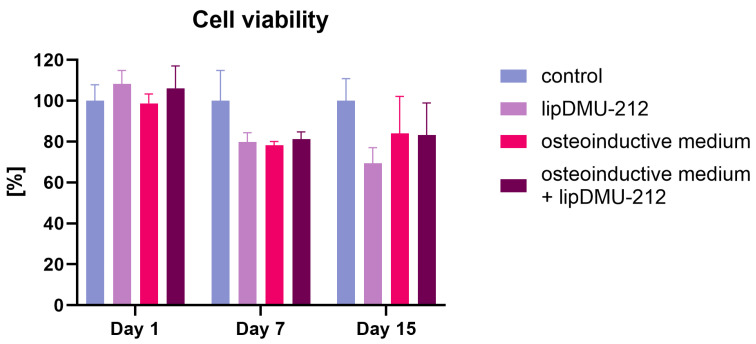
The effect of osteoinductive medium, lipDMU-212 at the concentration of 5 μM, or the combination of osteoinductive medium with lipDMU-212 at the concentration of 5 μM on hGCs spheroids, compared with the control (culture medium), after 1, 7, and 15 days of culture. The results of three independent replicates are presented as means ± SDs.

In light of our prior research [19], the liposomal formulation of DMU-212 at a concentration of 5 μM was selected for the differentiation studies. The results of the cell viability assay indicated no significant alterations in the viability of hGC spheroids following 24 h of exposure to an osteoinductive medium, lipDMU-212 at the concentration of 5 μM, or their combination (Figure 2). However, after 7 and 15 days of culture, a decrease in cell viability of approximately 20% was observed in all conditions tested. This decline is hypothesized to be associated with the cellular differentiation process.

### 2.3. Alizarin Red Staining

**Figure 3:**
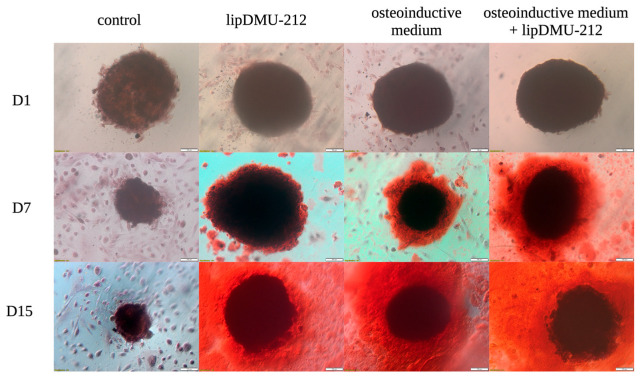
Osteogenic differentiation of hGCs spheroids after 1, 7, and 15 days of culture in culture medium (control), culture medium with lipDMU-212 at the concentration of 5 μM, osteoinductive medium, and a combination of osteoinductive medium with lipDMU-212 at the concentration of 5 μM. The red coloration indicates the presence of calcium in mineralized matrices. The scale bar in the image corresponds to 100 µm, 10× magnification.

The differentiation process of hGCs was confirmed by Alizarin Red staining, which is considered the gold standard for assessing osteoblast mineralization [20]. The results demonstrate that hGCs differentiated into osteoblasts when cultured in an osteoinductive medium, as well as in a culture medium containing lipDMU-212 at a concentration of 5 μM after 7 days of treatment. The most efficacious outcomes were observed with the combination of these two factors after 7 and 15 days of differentiation (Figure 3).

### 2.4. ALP Activity

**Figure 4:**
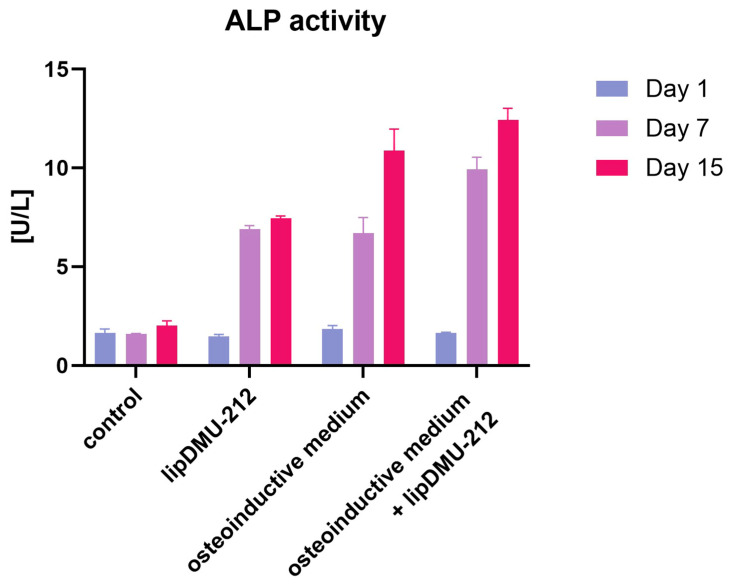
ALP activity [U/L] after 1, 7, and 15 days of hGCs culture in culture medium (control), culture medium with lipDMU-212 at a concentration of 5 μM, osteoinductive medium, and a combination of osteoinductive medium with lipDMU-212 at a concentration of 5 μM. The results of three independent replicates are presented as the means ± SDs.

The differentiation process was quantified by measuring ALP activity. The results indicated a significant increase in ALP activity following 7 and 15 days of culture in the osteoinductive medium, treatment with lipDMU-212 at the concentration of 5 μM, and the osteoinductive medium with lipDMU-212 combined. Following 7-day treatment, a threefold increase in ALP activity was observed in samples treated with the osteoinductive medium and lipDMU-212 at a concentration of 5 μM. The most pronounced effects were observed following the 7 and 15 culture periods in the osteoinductive medium with lipDMU-212 at a concentration of 5 μM (Figure 4).

### 2.5. RNA-Seq

RNA-seq analysis was performed to explore the mechanism underlying the role of lipDMU-212 in osteogenic differentiation. The analysis was performed following 15 days of treatment of hGC spheroids with lipDMU-212 at a concentration of 5 μM, and with the osteoinductive medium used as a comparison. RNA-seq reveals that lipDMU-212 promotes cell differentiation and cellular identity.

**Figure 5:**
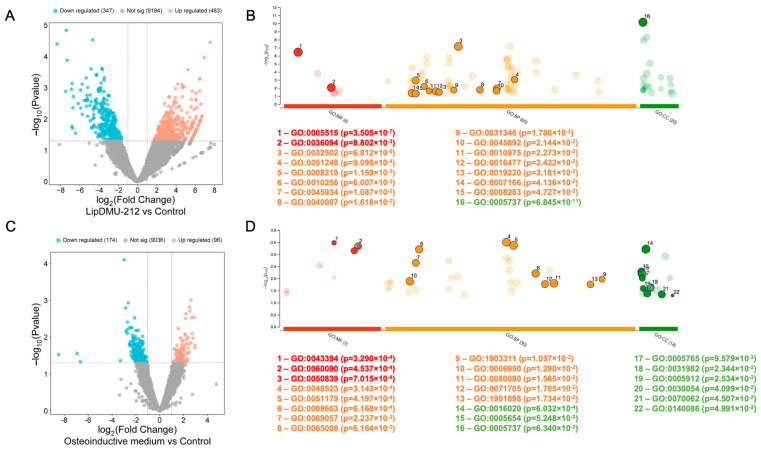
Gene expression analysis of hGC spheroids after treatment with lipDMU-212 and osteoinductive medium. (**A**) Volcano plot of gene expression profiles for lipDMU-212 treatment in hGC spheroids. (**B**) Gene Ontology enrichment analysis of DEGs of hGC spheroids after lipDMU-212 treatment. (**C**) Volcano plot of gene expression profiles for osteoinductive medium treatment in hGC spheroids. (**D**) Gene Ontology enrichment analysis of DEGs of hGC spheroids after osteoinductive medium treatment in hGC spheroids.

Differential expression analysis revealed 830 DEGs in hGCs, with 483 up-regulated and 347 down-regulated DEGs (Figure 5A). lipDMU-212 enhanced the expression of genes involved in coordinated cellular reprogramming response in hGCs, potentially involving Wnt pathway modulation; membrane reorganization; developmental signaling cascades that facilitate osteogenic differentiation, such as *GATA4, PTMAP5*, *NKD1*, *PI4K2B*, *NTRK3*, *CNTNAP3*, *CCDC187*, and *TRAPPC2L*; and developmental signaling cascades that weaken the expression of developmental and differentiation control genes, such as *MEI4*, *NPY4R*, and *CLDN24*, and non-coding RNA elements such as the antisense RNAs *PAPPA-AS2*, *DENND2B-AS1*, *SOX5-AS1*, *SLC1A2-AS2*, and *MAGI1-AS1* and the pseudogenes *RPL6P12*, *PAFAH1B1P2*, *ELOA3P*, *TPM3P1*, *OR7E13P*, *RPL6P7*, and *ZBTB45P1.* In addition, Gene Ontology (GO) and Kyoto Encyclopedia of Genes and Genomes (KEGG) analyses were performed to obtain deeper insights into signaling pathways induced by lipDMU-212 in hGCs. GO enrichment analysis showed that all the DEGs were enriched into 91 items: 65 biological processes, 20 cellular components, and 6 molecular functions. Highly expressed functions in the hGCs included “developmental process”, “negative regulation of protein metabolic process”, “cell death”, “endomembrane system organization”, “negative regulation of nucleobase-containing compound metabolic process”, “negative regulation of nucleobase-containing compound metabolic process”, “negative regulation of DNA-templated transcription”, “regulation of phosphate metabolic process”, “growth”, “cell migration”, “protein binding”, “small molecule binding”, “cell population proliferation”, and “regulation of phosphate metabolic process” (Figure 5B).

In the case of the osteoinductive medium, the differential expression analysis revealed 270 DEGs in hGCs, with 96 up-regulated and 174 down-regulated DEGs (Figure 5C). The osteoinductive medium enhanced the expression of genes involved in key osteogenic signaling, structural remodeling, and neural crest-like reprogramming such as *SMAD3*, *RAP2C*, *KRT18*, *GPLD1*, and *CNTN2* and weakened the expression of extracellular matrix, metabolic regulation, and RNA processing genes, such as *AGRN*, *ADAMTS9-AS1*, *MIR663AHG*, *FCMR*, *CELF2-AS2*, *SLC7A5*, and *EBP2.*

GO enrichment analysis showed that all the DEGs were enriched into 51 items: 30 biological processes, 14 cellular components, and 7 molecular functions. Highly expressed functions in the hGCs included “cell adhesion molecule binding”, “molecular adaptor activity”, “proteoglycan binding”, “negative regulation of cellular process”, “anatomical structure morphogenesis”, “localization”, “macromolecule catabolic process”, “regulation of mRNA metabolic process”, “regulation of biological quality”, “response to stress”, “regulation of primary metabolic process”, “basement membrane/interstitial matrix interface”, “extracellular exosome”, “cell junction”, “adherens junction”, “lysosomal membrane”, “membrane”, “cytoplasm”, “nucleoplasm”, “vesicle”, “nitrogen compound transport”, and “regulation of primary metabolic process” (Figure 5D).

### 2.6. Differential Modulation of the Wnt Pathway by lipDMU-212 and Osteoinductive Medium

**Figure 6:**
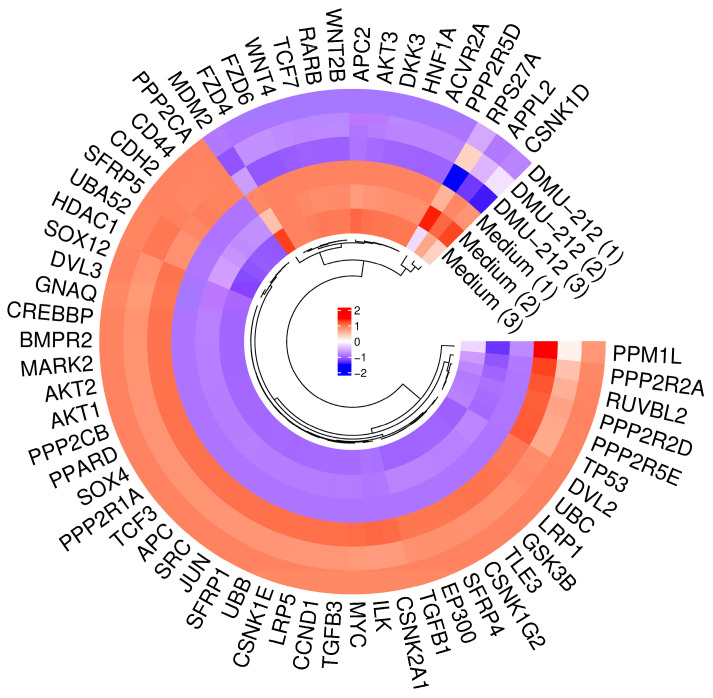
Heatmap of differential expression genes (DEGs) of hGCs spheroids after lipDMU-212 and osteoinductive medium treatment related to Wnt pathway.

The expression of DEGs enriched in the Wnt-related pathway is presented in a heatmap (Figure 6). The comparative transcriptomic analysis revealed contrasting patterns of Wnt pathway regulation following treatment of hGCs with lipDMU-212 versus the osteoinductive medium.

lipDMU-212 treatment predominantly led to up-regulation of Wnt pathway components, with seven genes reaching statistical significance. Among these, *JUN*, *CSNK2A1*, and *LRP1* were most notable, reflecting both the activation of transcriptional machinery (*JUN*) and the reinforcement of receptor-mediated signaling (*LRP1*).

In contrast, the osteoinductive medium induced a pattern of predominant down-regulation within the Wnt pathway, with three genes showing significant changes. Notably, *APC2* was up-regulated, while *LRP1* and *SFRP4* were significantly down-regulated, suggesting a more selective modulation of the pathway.

## 3. Discussion

The results of our studies indicate that the liposomal formulation of DMU-212 promotes osteogenic differentiation of hGCs in a 3D primary cell culture model. Both qualitative assessment using Alizarin Red staining and quantitative measurement of ALP activity demonstrated enhanced mineralization and induction of an osteoblastic phenotype in hGCs in the presence of lipDMU-212. This effect was particularly pronounced when lipDMU-212 was combined with an osteoinductive medium, suggesting a synergistic effect of both factors.

In the first stage of the study, a liposomal formulation of DMU-212 was developed to address its poor water solubility, enhance its dispersion in an aqueous environment, and prevent precipitation. Liposomes represent an effective tool in pharmaceutical drug delivery, as they increase the bioavailability of hydrophobic compounds and provide protection from degradation [21]. Moreover, the liposomal formulation of the compound may further enhance its bioavailability and, consequently, its biological activity, as previously demonstrated [18].

Furthermore, a spheroid model of hGCs was employed in our study, since 2D cell monolayers have many limitations and do not entirely mimic in vivo features. Compared with 2D cultures, the 3D model more closely reflects in vivo conditions, providing benefits such as the preservation of appropriate cell polarity, cell-to-cell interactions, and a proper distribution of nutrients and oxygen, as well as gene and protein expression patterns that better reflect those observed in living tissues. This approach allowed for a more accurate assessment of the biological activity of lipDMU-212 under conditions resembling the native cellular microenvironment [22].

The cytotoxicity assay was performed to ensure the applied compound’s safety for differentiation studies. Based on our previous research [19], we selected a concentration of 5 μM for further research. lipDMU-212 did not significantly affect hGC spheroid viability after 24 h, either alone or in combination with the osteoinductive medium. The ~20% reduction in viability observed after 7 and 15 days in all experimental groups likely reflects the progression of cellular differentiation [23].

hGCs are known to display mesenchymal stem cell-like characteristics. To date, hGCs have been reported to differentiate into multiple cell lineages in long-term in vitro culture. Kossowska-Tomaszczuk et al. demonstrated that hGCs are able to differentiate into osteogenic, chondrogenic, and adipogenic lineages in long-term culture, particularly in the presence of LIF [4]. In this context, hGCs represent a promising source for generating osteoblast populations, which might be effectively applied in regenerative medicine to treat bone-related disorders. In our studies, the osteogenic differentiation of hGCs was carried out for 15 days. To verify the ability of lipDMU-1212 to induce hGC differentiation, an osteoinductive medium was used as a positive control. The differentiation of hGCs was confirmed by Alizarin Red staining, which remains the gold standard for evaluating osteoblast mineralization. Alizarin Red staining involves the chelation of calcium ions by anthraquinone dye to form a bright red complex, which can be visualized microscopically and quantified by subsequent colorimetric analysis [20]. hGCs cultured either in the osteoinductive medium or in the medium containing lipDMU-212 at 5 μM displayed signs of osteoblastic differentiation after 7 and 15 days, with the most pronounced effects being observed when both factors were combined. These results were confirmed and quantified by ALP activity measurements, which revealed significant increases at both 7 and 15 days across all treatment groups. Notably, a threefold increase in ALP activity was noted after 7 days in cultures treated with the osteoinductive medium and lipDMU-212 in combination, indicating a synergistic effect on the early stages of osteogenic differentiation.

Furthermore, we performed RNA-seq analysis to explore the mechanism underlying the role of lipDMU-212 in osteogenic differentiation. The analysis was undertaken following 15 days of exposure of hGC spheroids to 5 μM lipDMU-212 and to the osteoinductive medium, compared with the control (culture medium). Numerous findings have demonstrated that resveratrol is able to induce the differentiation of MSCs. Previous studies showed that resveratrol promotes osteogenesis while inhibiting adipogenesis in human embryonic stem cell-derived mesenchymal progenitors. These effects were mainly mediated through the SIRT1/FOXO3A signaling pathway, which enhanced the expression of osteogenic genes (*RUNX2* and *osteocalcin*) [24]. On the other hand, resveratrol has been shown to induce osteogenic differentiation in canine bone marrow MSCs via activation of the Wnt/β-catenin and ERK/MAPK signaling pathways [25].

Similarly, the obtained results indicated that lipDMU-212 enhanced the expression of genes involved in cellular reprogramming response in hGCs. This coordinated down-regulation of regulatory non-coding RNAs, and cell-type-specific genes represents a systematic dismantling of hGC identity, creating a permissive chromatin environment for osteogenic transcription factors to establish new gene expression programs.

In the context of functional implications, this indicates early-stage dedifferentiation—removal of cell-type-specific regulatory constraints—and represents the “erasing” phase of transdifferentiation. We also observed up-regulation of *GATA4*, a zinc-finger transcription factor that plays an important role in osteoblast differentiation. GATA4 is known to regulate osteoblast function by modulating the balance between TGFβ and BMP signaling pathways [26]. GATA4 has additionally been identified as a regulator of osteoblasts, functioning by modulating estrogen receptor recruitment to chromatin in a tissue-specific context [27].

In the osteogenic context, it points to epigenetic derepression and loss of granulosa identity. In summary, it can be stated that lipDMU-212 causes extensive epigenetic reprogramming, creating cellular plasticity. DMU-212 could prime cells for enhanced responsiveness to osteoinductive signals by (I) opening chromatin (H19, MALAT1, and YEATS2), (II) enhancing cellular plasticity (RND3 and ion channels), and (III) activating stemness programs by lncRNA networks. Additionally, RNA-seq data revealed an up-regulation of genes associated with osteoblast lineage markers, including *COL1A2*, *COL6A1*, and *COL6A2.* These findings are consistent with Brązert et al.’s, who also reported an increased expression of osteoblast marker genes (e.g., *COL1A1*) following long-term differentiation of hGCs [28]. These genes encode major components of type I collagen, essential to bone matrix formation, and type VI collagen, which builds extracellular microfibrils, contributing to osteoblast function and extracellular matrix organization [29,30]. Furthermore, we noticed the up-regulation of *LARP6*, which is involved in the regulation of type I collagen formation by modulating the stability of collagen mRNA [31].

Interestingly, RNA-seq data also revealed significant up-regulation of Wnt-related genes, suggesting that lipDMU-212 but also the osteoinductive medium may potentially modulate the Wnt/β-catenin signaling pathway. Several genes of clinical and mechanistic interest were identified. *JUN* demonstrated the most striking divergence, showing robust up-regulation under lipDMU-212 treatment (+2.82, *p* = 0.003) but down-regulation under osteoinductive conditions (−1.40, *p* = 0.061). *JUN*, as a part of the AP-1 transcription factor complex, contributes to the transcriptional regulation of osteoblast-specific genes, e.g., *Runx2* and collagen-related genes, thereby promoting osteoblast differentiation. *LRP1,* which acts as a multifunctional receptor and plays a crucial role in endocytosis and cellular signaling, was significantly regulated by both treatments, though in opposing directions, underscoring its pivotal role in Wnt signaling and cell fate determination [32].

Another up-regulated gene after lipDMU-212 treatment was *AXIN1*, which, on the other hand, coordinates interactions with proteins such as GSK-3β, CK1, APC, and β-catenin, enabling phosphorylation and proteasomal degradation of β-catenin [33]. Upon Wnt ligand (e.g., Wnt1, Wnt3a, and Wnt8) binding to Frizzled and LRP5/6 receptors, AXIN1 is recruited to phosphorylated LRP5/6, which suppresses GSK-3β activity within the destruction complex and consequently stabilizes β-catenin [34]. Because AXIN1 is present at relatively low levels compared with other complex components, it serves as a concentration-limiting factor and a key regulator of Wnt/β-catenin signaling. *FYN* is a member of the SRC family of protein tyrosine kinases, which mediates key signaling downstream of integrin receptors and growth factor pathways that intersect with Wnt/β-catenin signaling [35]. Activation of Wnt/β-catenin enhances expression of master osteogenic factors such as RUNX2 and Osterix and promotes extracellular matrix mineralization [36]. Finally, *APC2* was uniquely up-regulated in response to the osteoinductive medium, which indicates that it may act as a specific mediator of this treatment’s effects.

Collectively, these findings highlight that lipDMU-212 and osteoinductive medium elicit distinct yet potentially complementary influences on Wnt signaling and osteogenic differentiation of hGCs. This divergence provides a rationale for exploring combination strategies to harness synergistic effects in regenerative or therapeutic contexts.

On the other hand, treatment of hGC spheroids with an osteoinductive medium leads to the remodeling of cellular structure and metabolic functions. The up-regulation of *SMAD3* indicates activation of crucial osteogenic signaling pathways that regulate mesenchymal stem cell differentiation into osteoblasts [37]. This represents the core molecular switch driving osteogenesis. The presence of *CNTN2* suggests that granulosa cells may be adopting neural crest-like characteristics, which is significant because neural crest cells have high osteogenic potential and share developmental origins with many mesenchymal tissues [38]. *KRT18* up-regulation indicates cytoskeletal remodeling necessary for the morphological changes accompanying osteoblast differentiation [39]. Compared with lipDMU-212, the osteoinductive medium activates classical osteogenic differentiation via SMAD3/BMP signaling and, in the context of functional implications, represents mid-stage remodeling—the dismantling of existing cellular infrastructure and metabolic pathways. In the osteogenic context, it points to structural and metabolic reorganization to support osteoblast differentiation.

There is substantial evidence in the literature supporting the pro-differentiation effects of resveratrol and its derivatives on cells exhibiting mesenchymal characteristics. However, to the best of our knowledge, this is the first study to demonstrate the ability of DMU-212 to promote osteogenic differentiation.

## 4. Materials and Methods

### 4.1. Chemicals and Reagents

All chemicals used were of analytical grade unless specified otherwise. DMU-212 was purchased from Sigma-Aldrich Co. (St. Louis, MO, USA). 1-Palmitoyl-2-oleoyl-sn-gly-cero-3-phosphocholine (POPC) and 1-palmitoyl-2-oleoyl-sn-glycero-3-phospho-(1′-rac-glycerol) (sodium salt) (POPG) were obtained from Avanti Polar Lipids (Alabaster, AL, USA). Chloroform and acetonitrile were acquired from Avantor Performance Materials Inc. (Radnor, PA, USA). The CellTiter-Glo^®^ 3D Cell Viability Assay Kit was supplied by Promega Co. (Madison, WI, USA). All additional materials, unless mentioned otherwise, were acquired from Sigma-Aldrich Co. (St. Louis, MO, USA).

### 4.2. Liposome Formulation and Characterization

Liposomes were formulated using the thin-film hydration (TLH) method, followed by extrusion, as described elsewhere [19]. To determine liposome size and zeta potential, dynamic light scattering (DLS) and laser Doppler electrophoresis (Malvern Zetasizer Nano ZS, Malvern Instruments Ltd., Malvern, UK) were used. The concentration of DMU-212 in the liposomal formulation was analyzed by High-Performance Liquid Chromatography (HPLC) at 40 °C. Liposome samples were diluted fivefold in methanol before analysis, performed on an Agilent 1260 Infinity II system (Agilent Technologies, Santa Clara, CA, USA) equipped with a DAD. Chromatographic separation was performed using a Luna Omega PS C18 column (150 mm × 4.6 mm I.D., 3 μm particle size) (Phenomenex Inc., Torrance, CA, USA). The mobile phase consisted of eluent A (water, 5 mM ammonium acetate, and 0.1% formic acid) and eluent B (acetonitrile), applied at a flow rate of 0.8 mL/min. The gradient started at 60% A for 3 min, gradually decreased to 10% A over 4 min, held at 10% A for 2 min, and then increased back to 60% A within 1 min, followed by a 5 min re-equilibration. DMU-212 was detected at 325 nm, with a retention time of 9.7 min. Data acquisition and processing were performed using OpenLAB CDS ChemStation Edition Rev. C.01.07 SR3.

Encapsulation efficiency (EE, %) was calculated using the formula

EE = (Cm/Ci) × 100%

where Cm—DMU-212 concentration loaded into liposomes (determined by HPLC); Ci—theoretical maximum concentration at 100% efficiency.

### 4.3. Source and Culture of Human Ovarian Granulosa Cells

hGCs were obtained from follicular fluid collected from 20 women aged 25–40 years undergoing IVF-ICSI procedures due to tubal or male factor infertility. The samples were gathered at the Center for Gynecology, Obstetrics, and Infertility Treatment Pastelova in Poznan, Poland. Follicular fluid was retrieved via transvaginal ultrasound-guided aspiration following controlled ovarian stimulation according to a predefined protocol, as previously described [40]. After removing the cumulus–oophorus–oocyte complexes, the freshly acquired follicular fluid was centrifuged at room temperature for 10 min at 250× *g*. The GCs were further purified using density gradient centrifugation. Specifically, the cell pellet was resuspended in Phosphate-Buffered Saline (PBS) and layered onto 7 mL of Pancoll human (PAN-Biotech, Berlin, Germany). hGCs were further separated using density gradient centrifugation with Pancoll human for 20 min at 400× *g*. The GCs were aspirated from the interphase layer, washed in 10 mL of Dulbecco’s Modified Eagle Medium (DMEM), centrifuged at 250× *g* for 10 min, and resuspended in a cell culture medium. The isolated cells were seeded into cell culture flasks and initially cultured for 24 h in DMEM supplemented with 10% Fetal Bovine Serum (FBS), 10 mg/mL gentamicin, 10,000 μg/mL streptomycin, 10,000 U/mL penicillin, and 4 mM L-glutamine [40]. Culturing was performed at 37 °C in a humidified atmosphere of 5% CO_2_ and 95% air. After 24 h, the cells were detached using a trypsin–EDTA solution and seeded into 96-well U-bottom plates at a density of 10,000 viable cells per well. To promote spheroid formation, cells were transferred to a culture medium containing methylcellulose at a final concentration of 0.25% [41], centrifuged at 280× *g* for 10 min, and incubated for 72 h. Spheroids were measured to ensure uniform size, with an average diameter of 374.62 ± 19.45 μm used for the experiments.

### 4.4. Cell Viability Assay

The CellTiter-Glo^®^ 3D Cell Viability Assay was employed to assess the impact of lipDMU-212 on GC spheroids. After 72 h of spheroid growth, lipDMU-212, osteoinductive medium, or osteoinductive medium with lipDMU-212 were added at a concentration of 5 μM. The spheroids were treated with the test compounds for 1, 7, and 15 days, after which the assay was conducted following the manufacturer’s instructions. Specifically, an equal volume of reagent was added to both treated and untreated GC spheroids, followed by 30 min of incubation. The resulting lysates were then transferred to white 96-well plates, and luminescence was measured using a Tecan Infinite 200 Pro reader (Tecan Austria GmbH, Grödig, Austria).

### 4.5. Alizarin Red Staining

The Alizarin Red staining was performed to qualitatively characterize the mineralization process. After 1, 7, and 15 days of culture in culture medium (control), culture medium with lipDMU-212 at the concentration of 5 μM, osteoinductive medium, and a combination of both, spheroids were rinsed three times with PBS and fixed with Saccomanno fixing solution (Morphisto, Hessen, Germany) for 30 min. Then, spheroids were washed with distilled water, and Alizarin Red solution (Sigma-Aldrich, Saint Louis, MO, USA) was added for 15 min at room temperature. The excess dye was removed, and spheroids were washed using deionized water. Calcium deposition was assessed based on the red color intensity.

### 4.6. ALP Activity Assessment

The differentiation of hGCs into osteoblasts was quantitatively assessed using an ALP assay kit (Abcam Inc., Cambridge, UK) following the manufacturer’s instructions. After spheroid formation, hGCs were maintained under four different conditions: medium (control), medium supplemented with 5 μM lipDMU-212, osteoinductive medium, and a combination of lipDMU-212 and osteoinductive medium. After 1, 7, and 15 days of culture, cell lysates were collected, and ALP levels were measured according to the manufacturer’s protocol. Absorbance was measured at 450 nm using a microplate reader (Tecan Infinite 200 Pro, Männedorf, Switzerland).

### 4.7. RNA Isolation

RNA was isolated from hGC spheroids after 1 day (early control) and 15 days of culture. Cells were grown under three conditions: culture medium, medium supplemented with 5 μM lipDMU-212, or osteoinductive medium. For RNA isolation, spheroids were collected, washed with PBS, and immediately lysed in 0.5 mL of TRIzol reagent (Thermo Fisher Scientific, Waltham, MA, USA). Samples were then stored at −80 °C until further processing. RNA extraction was performed using the TRIzol Plus Purification Kit (Thermo Fisher Scientific) according to the manufacturer’s instructions. The quantity and purity of isolated RNA were assessed by measuring the optical density at 260 nm, and purity was evaluated by the 260/280 nm absorption ratio using a NanoDrop 2000 spectrophotometer (Thermo Fisher Scientific). Only samples exhibiting a 260/280 ratio above 1.8 were used for subsequent analyses.

### 4.8. Library Preparation and RNA Sequencing

About 300 ng of total RNA was used for library preparation with the KAPA RNA HyperPrep Kit with RiboErase (HMR) (Illumina Platform, San Diego, CA, USA). Adaptor ligations were performed according to the manufacturer’s instructions, and cDNA fragments were amplified in RT-qPCR for 9–15 cycles. After the purification with KAPA Pure Beads, the DNA concentration was measured with Qubit (Thermo Fisher Scientific, Waltham, MA, USA), and the fragment lengths were defined with Agilent 2100 Bioanalyzer (Agilent Technologies, Santa Clara, CA, USA). RNA sequencing was performed using Illumina NovaSeq X Series (Illumina, San Diego, CA, USA), with an average of ~60 million 150 paired-end reads per sample.

## 5. Conclusions

This study demonstrates that lipDMU-212 effectively promotes osteogenic differentiation of hGCs in a 3D cell culture model. RNA-seq analysis revealed that lipDMU-212 induces extensive transcriptional reprogramming characterized by epigenetic derepression, loss of granulosa identity, and activation of osteogenic lineage-specific genes. Interestingly, the study revealed that lipDMU-212 and the osteoinductive medium may engage mechanistically distinct routes of osteogenic differentiation: lipDMU-212 likely exerts its effect via Wnt pathway activation, whereas the osteoinductive medium appears to promote osteogenesis through alternative pathways while concurrently suppressing specific WNT elements. Given the multipotent nature of hGCs and their accessibility, lipDMU-212 may represent a promising tool in regenerative medicine strategies targeting bone repair and treatment of degenerative skeletal disorders.

## Data Availability

The original contributions presented in this study are included in the article. Further inquiries can be directed to the corresponding author.
